# Translational model of melphalan-induced gut toxicity reveals drug-host-microbe interactions that drive tissue injury and fever

**DOI:** 10.1007/s00280-021-04273-7

**Published:** 2021-04-20

**Authors:** H. R. Wardill, C. E. M. de Mooij, A. R. da Silva Ferreira, I. P. van de Peppel, R. Havinga, H. J. M. Harmsen, W. J. E. Tissing, N. M. A. Blijlevens

**Affiliations:** 1 Adelaide Medical School, The University of Adelaide, Adelaide, South Australia Australia; 2 Department of Pediatrics (Oncology and Hematology), University of Groningen, University Medical Center Groningen, Groningen, The Netherlands; 3 Department of Hematology, Radboud University Medical Centre, Nijmegen, The Netherlands; 4 Department of Pediatrics (Molecular Metabolism and Nutrition), University of Groningen, University Medical Center Groningen, Groningen, The Netherlands; 5 Department of Medical Microbiology, University of Groningen, University Medical Center Groningen, Groningen, The Netherlands; 6 Princes Maxima Centre for Pediatric Oncology, Utrecht, The Netherlands

**Keywords:** Melphalan, Mucositis, Gut toxicity, Diarrhea, Infection, Microbiota

## Abstract

**Purpose:**

Conditioning therapy with high-dose melphalan (HDM) is associated with a high risk of gut toxicity, fever and infections in haematopoietic stem cell transplant (HSCT) recipients. However, validated preclinical models that adequately reflect clinical features of melphalan-induced toxicity are not available. We therefore aimed to develop a novel preclinical model of melphalan-induced toxicity that reflected well-defined clinical dynamics, as well as to identify targetable mechanisms that drive intestinal injury.

**Methods:**

Male Wistar rats were treated with 4–8 mg/kg melphalan intravenously. The primary endpoint was plasma citrulline. Secondary endpoints included survival, weight loss, diarrhea, food/water intake, histopathology, body temperature, microbiota composition (16S sequencing) and bacterial translocation.

**Results:**

Melphalan 5 mg/kg caused self-limiting intestinal injury, severe neutropenia and fever while impairing the microbial metabolome, prompting expansion of enteric pathogens. Intestinal inflammation was characterized by infiltration of polymorphic nuclear cells in the acute phases of mucosal injury, driving derangement of intestinal architecture. Ileal atrophy prevented bile acid reabsorption, exacerbating colonic injury via microbiota-dependent mechanisms.

**Conclusion:**

We developed a novel translational model of melphalan-induced toxicity, which has excellent homology with the well-known clinical features of HDM transplantation. Application of this model will accelerate fundamental and translational study of melphalan-induced toxicity, with the clinical parallels of this model ensuring a greater likelihood of clinical success.

**Graphic abstract:**

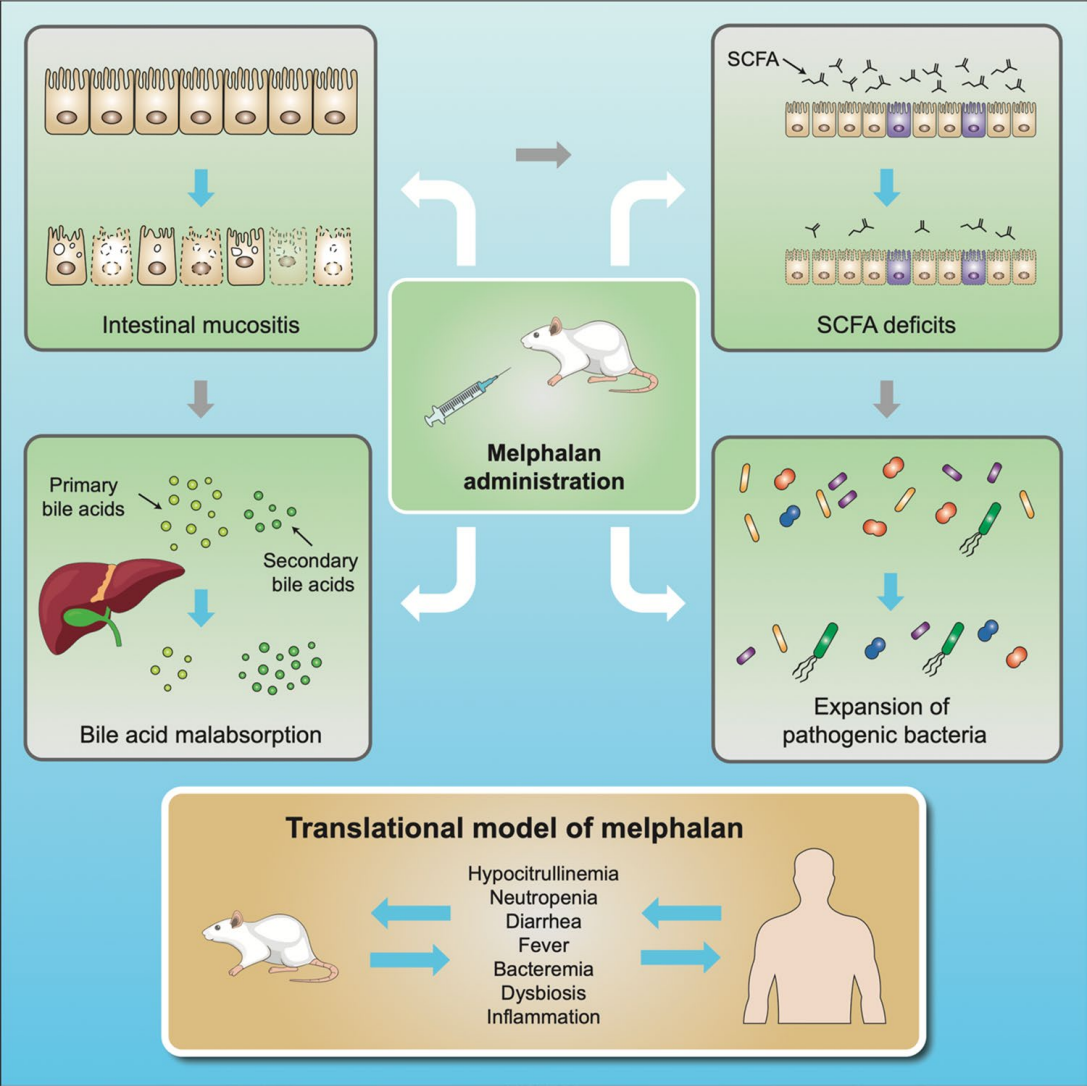

**Supplementary Information:**

The online version contains supplementary material available at 10.1007/s00280-021-04273-7.

## Introduction

Melphalan is an alkylating chemotherapeutic agent which has been in use for over 60 years and remains the cornerstone of successful multiple myeloma (MM) treatment, even in the era of novel agents [1, 2]. While high-dose melphalan (HDM, 200 mg/m^2^) is associated with optimal efficacy and survival [3], it is associated with significant toxicity, which currently limits its use in less fit and elderly patients. HDM induces considerable oral and gut toxicity (mucositis), and febrile neutropenia is seen in approximately 80–85% of patients [4, 5].

Breakdown of the mucosal barrier has been identified as a key risk factor for bacteremia [6, 7], permitting translocation of enteric pathogens into systemic circulation. While largely based on clinical observation, these findings strongly implicate acute gastrointestinal injury in the etiology of bacteremia and suggest that promoting the integrity of the mucosal barrier may control infection risk while simultaneously reducing use of antimicrobial agents and limiting disruption of the microbiota (dysbiosis). In turn, this would minimize deleterious effects of antibiotics and dysbiosis, which are increasingly reported to decrease overall survival [8–11], worsen acute and chronic toxicities [12–15] and drive the emergence of antibiotic-resistant strains [16].

Unfortunately, there remain no effective prophylactic or therapeutic interventions for HDM-induced gut toxicity [17]. In fact, despite demonstrated efficacy in other oncological cohorts, palifermin fails to have any appreciable impact in patients receiving HDM [18]. Our current inability to optimally prevent or manage HDM-induced gut toxicity largely reflects a limited understanding of its core pathobiology. Clinically, the dynamics of mucosal injury caused by HDM are well-described, particularly relating to the longitudinal changes in plasma citrulline—a validated biomarker of small intestinal enterocyte mass [5, 19, 20]. However, the unique molecular mechanisms that dictate the clinical phenotype are challenging to dissect given the practical obstacles in conducting invasive investigations in this highly vulnerable patient cohort. This is further compounded by the complete lack of translationally robust preclinical models.

To date, several preclinical studies have used melphalan-based models [21] to evaluate interventions/mechanisms including epidermal growth factors [22] and caspase-11 [23]. However, these models are mostly outdated, do not report key toxicity outcome measures and lack synergy with the clinical dynamics of HDM transplantation. As such, there is a clear disconnect between the clinical understanding of HDM-induced gut toxicity and our ability to identify targetable mechanisms in a preclinical setting, hindering efforts to develop interventions that successfully translate to the clinic [24]. The current study therefore aimed to develop and characterize a new preclinical model of HDM-induced gut toxicity and identify candidate pathways for therapeutic intervention.

### STAR methods

This study is reported using the ARRIVE guidelines for the accurate and reproducible reporting of animal research and the STAR methods for structured, transparent and accessible reporting.

### Experimental model details

#### Ethical statement and animal husbandry

All animal studies were conducted in accordance with the ethical guidelines approved by the Dutch Centrale Commissie Dierproeven (CCD) and the Institutional Animal Care and Use Committee of the University Medical Center Groningen, University of Groningen (RUG), under the License Number 171325-01. All animals were individually housed in conventional, open cages at the Centrale Dienst Proefdieren (CDP; Central Animal Facility) at the University Medical Center Groningen. Rats were housed under 12 h light/dark cycles with ad libitum access to autoclaved AIN93G rodent chow and sterile water. Sawdust bedding was provided in all cases as well as a toilet roll for enrichment. All cages were randomly arranged across racks to prevent potential bias.

#### Study design

Animal experiments were performed as an initial dose finding study (4–8 mg/kg melphalan, sourced from the Pharmachemie Holding, B.V. The Netherlands), in which body weight, diarrhea, food/water intake and plasma citrulline were used to determine the optimal dose of melphalan in which moderate, self-limiting mucositis was achieved (based on citrulline dynamics). A dose validation study was then performed using the optimized dose of melphalan (5 mg/kg) for which all endpoint analyses were performed (*N* = 24 vehicle, *N* = 24 melphalan). In both cases, male Wistar rats weighing between 80 and 100 g were allowed to acclimatize for 10 days after arriving at the CDP. Rats were then randomized to receive 4 (*N* = 6), 5 (*N* = 6 dose finding, *N* = 24 dose validation), 6 (*N* = 6) or 8 mg/kg (*N* = 3) melphalan (10 mg/ml), or a volume equivalent dose of vehicle solution (0.9% NaCl, *N* = 3 dose finding, *N* = 24 dose validation). All intravenous injections were performed via the penile vein under anesthetic (3% isoflurane) on day 0. Minor adjustments were made to random allocation to ensure experimental groups exhibited comparable baseline body weight.

#### Melphalan treatment and tissue preparation

Rats received a single intravenous injection of melphalan (10 mg/ml, 4–8 mg/kg) or vehicle solution on day 0 of the experimental time course (Figure S1). Melphalan or vehicle control was administered via the penile vein at 10 am in designated procedure rooms within the CDP. For initial dose finding studies, all rats were sacrificed via isoflurane anesthesia, cardiac puncture and cervical dislocation at day 10 (recovery). For dose validation studies (5 mg/kg), groups of rats were followed longitudinally until day 10 (*N* = 8/group). Subsets of rats were sacrificed on day 4 (*N* = 4/group) and day 7 (*N* = 4/group) for exploratory investigation.

At sacrifice, the liver, spleen, kidneys and cecum were weighed, and intestinal lengths recorded. All organs were drop-fixed in 10% neutral buffered formalin before being processed and embedded in paraffin. Cecal contents were snap frozen in liquid nitrogen. Intestinal resections were flushed with ice-cold, sterile 1 X phosphate buffered saline (PBS) and 1 cm sections were prepared for fixation in 10% neutral buffered saline, snap frozen in liquid nitrogen.

The primary outcome of the study was plasma citrulline, a validated biomarker of mucosal injury [19, 20, 25–27]. Secondary outcomes included survival, weight loss, diarrhea, white blood cell counts, body temperature, peripheral cytokines, blood cultures, intestinal barrier function, intestinal morphology (determined histopathologically), microbiome composition, short chain fatty acid (SCFA) profiles and plasma bile acids.

### Method detail

#### Clinical toxicity assessment

Systemic toxicity was assessed using clinical parameters of body weight, food intake and water intake, as well as routine welfare indicators (reluctance to move, posture, coat condition). Rats were weighed daily, and water/food intake monitored by manual weighing of chow and water bottles. Gut toxicity was determined by diarrhea severity assessed using a validated, semi-quantitative scoring system (0–3) where 0 = no diarrhea, 1 = mild diarrhea with soft stools and perianal staining, 2 = moderate diarrhea with loose stools and perianal staining of fur, 3 = severe diarrhea with watery stools with or without mucous, and fur staining incorporating the hind legs. This assessment scale has been used in both rats [28] and mice [29] treated with chemotherapy.

#### Plasma citrulline

Plasma citrulline is an indicator of small intestinal enterocyte mass, and a validated biomarker of small intestinal toxicity [19, 20]. Repeated blood samples (75 μl) were collected from the tail vein into EDTA-treated hematocrit capillary tubes on day 0, 1, 2, 3, 4, 6, 8 and 10 (for dose finding studies) and days 0, 2, 4, 6, 7, 8 and 10 for dose validation studies (between 8:00 and 10:00 am). Citrulline was determined in 30 μl of plasma (isolated from whole blood via centrifugation at 4000*g* for 10 min) using automated ion exchange column chromatography as previously described^10^.

#### Differential blood analysis

Whole blood samples (200 μl) were collected into MiniCollect^®^EDTA tubes from the tail vein of all rats at the time of treatment (day 0) and time of termination (Day 4, 7, 10). Differential morphological analysis included (10^9^/L and % unless defined otherwise): white blood cell count (WBC), red blood cell count (RBC), hemoglobin (HGB, mmol/L), hematocrit (HCT, L/L), mean corpuscle volume (MCV, fL), platelet count (PLT, 10^9^/L), nucleated red blood cell (NRBC) and WBC differentials (neutrophils, lymphocytes, monocytes, eosinophils, basophils). Differential morphological analyses were performed using routine protocols in the diagnostics suite (Dept Hematology) at the University Medical Centre Groningen.

#### Body temperature

Core body temperature was used as an indicator of fever and was assessed daily using the Plexx B.V. DAS-7007R handheld reader and IPT programmable transponders. Transponders were inserted subcutaneously under mild 2% isoflurane anesthesia on day− 4 (Figure S1). Body temperature was assessed once daily between 8:00 and 10:00 am. Average values from day − 4 to − 1 were considered as baseline body temperature.

#### Intestinal barrier function

Intestinal barrier function was assessed using 4 kDa fluorescein isothiocyanate (FITC)-dextran (Sigma-Aldrich) as previously described [30]. Briefly, FITC-dextran was prepared at a concentration of 120 mg/ml in sterile 1 X PBS (pH 7.4) and kept on ice, protected from light until administration. FITC-dextran (500 mg/kg) was administered to rats via oral gavage 3 h prior to termination. Administration was staggered in 15 min intervals to account for the time taken to terminate each animal and collect biospecimens. FITC-dextran concentrations were determined in plasma relative to a standard curve (0.0001–10 μg/ml) using the BioTek Synergy Mx Microplate Reader and Gen5 software.

#### Histology

Routine hematoxylin and eosin (H and E) staining was performed on jejunum, ileum and colon segments to evaluate intestinal architecture. Briefly, drop-fixed tissue was processed and embedded into paraffin wax. 4 μm sections were cut on a rotary microtome and mounted onto glass Superfrost^®^ slides. H and E staining was performed as per routine protocols and slides were scanned using the Hamamatsu Photonics Digital Slide Scanner (NanoZoomer S60). Images were evaluated in a blinded fashion using the NDP.view2 software. Villus height and crypt depth were measured using annotation tools in NDP.view2.10 well-oriented crypts/villi were measured per slide and an average calculated per animal.

Intestinal inflammation was determined by quantification of infiltrating polymorphic nuclear cells (PMNC) in the jejunum and colon, which were assessed in four regions (500 × 500 μm). Individual counts were averaged over the four regions to generate a single mean value for each specimen.

#### Blood culture

At termination, whole blood was collected via cardiac puncture and 2 ml was immediately dispensed into BD BACTEC^™^ PEDS Plus^™^/F plastic blood culture bottles. Bottles were stored at room temperature before being transported to the Medical Microbiology laboratory at the University Medical Centre. Culture bottles were incubated in the BD BACTEC^™^ Automated Blood Culture System for 96 h. Positive cultures were identified using matrix-assisted laser desorption/ionization-time of flight (MALDI-TOF) mass spectrometry (MS).

#### Microbiome analysis

Microbiota analysis was performed on repeated fecal samples collected on day 0, 4, 7 and 10 from *N* = 8 animals per group. Samples were collected aseptically and stored at − 20 °C. 16S rRNA Illumina sequencing was used to determine the composition of the fecal microbiota. All experimental procedures (DNA extraction, PCR amplification, PCR product quantification and pooling, purification, library preparation and sequencing) were performed by Novogene. For full methodology, please see ‘Supplementary methods’.

#### Short chain fatty acid analysis

Short chain fatty acids (SCFAs) were analyzed in cecal contents collected at termination. Briefly, the cecum was removed and the apex cut using sterile scissors. The contents were squeezed directly into a sterile 2 ml Eppendorf tube, snap frozen in liquid nitrogen and stored at − 80 °C. SCFAs were analyzed using gas chromatography adapted from Moreau et al. (2003) with minor adjustments [31]. Values were determined relative to a 7-point calibration curve (acetate: 0.0 and 8.0 mM; propionate: 0.0–4.0 mM; butyrate: 0.0–4.0 mM). Cecal contents were thawed on ice and diluted 1:9 with sterile, milliQ water and refrozen at − 80 °C. On the day of analysis, samples were thawed on ice and 500 µl was added to 500 μl of calibration sample, 100 μl internal standard solution (1 mg/ml 2-ethylbutyrate in Milli-Q), 20 μl 20% (w/v) sulphosalicylic acid solution and two drops 37% HCl were added. The sample was centrifuged at 16,100*g* for 20 min at 4 °C, and supernatant was transferred to a glass tube containing a spatula tip of sodium chloride. 2 ml of diethylether was added and the sample was vortexed for 10 min at room temperature and spun down at 3000*g* for 10 min at 4 °C. From the clear upper layer, a 500 μl aliquot was taken and transferred to a glass GC-vial. 50 μl of MBTSTFA + 1% TBDMCS was added and left to derivatize overnight at room temperature. 3 μl derivatized sample was injected into the GC–MS (7890A GC system and 5975C inert XI EI/CI MSD with an EI inert 350 source). Analysis was carried out in a split mode with an inlet split ratio of 20:1. Samples were analyzed in SIM acquisition mode; acetate at *m/z* 117, propionate at *m/z* 131, butyrate at *m/z* 145 and 2-ethylbutyrate at *m/z* 175. Injector, source and quadrupole temperatures were 280 °C, 230 °C and 150 °C, respectively. A Zebron capillary GC column of 30 m × 0.25 mm, 0.25 μm film thickness was used (ZB-1, Phenomenex, Torrance, USA). The GC oven was programmed as follows: 40 °C held for 0 min, increased to 70 °C at 5 °C min-1, held at 70 °C for 3.5 min, increase to 160 °C at 20 °C min-1, increased to 280 °C at 35 °C min-1 and finally held at 280 °C for 3 min with a total run time of 20.43 min. The flow was set a 1.0 ml min-1 with helium as carrier gas. Data processing was carried out with MassHunter Workstation Software (MassHunter, Agilent Technologies).

#### Bile acids and ileal gene expression

Bile acid profiles were determined in plasma isolated from whole blood collected at termination (by cardiac puncture). Plasma bile acids were quantified using an Ultra High Performance Liquid Chromatography system (SHIMADZU, Kyoto, Japan), coupled to a SCIEX QTRAP 4500 MD triple quadruple mass spectrometer (SCIEX, Framingham, MA, USA) (UHPLC-MS/MS) as previously described [32, 33]. Gene expression analysis was performed in on distal ileal segments, aligning with transporter expression, which were stored in RNAlater at − 20 °C. Total RNA was isolated using TRI-reagent (Sigma, St. Louis, MO, USA) and quantified by NanoDrop (NanoDrop Technologies, Wilmington, DE, USA). cDNA synthesis was performed from 1 µg of total RNA. Primers were designed with Primer-BLAST and optimized for use with SYBR Green Master Mix (Roche Diagnostics, Mannheim, Germany) (maximum product size 150 nucleotides). Real-time qPCR analysis was performed on a StepOnePlus^™^ Real-Time PCR System (Applied Biosystems, Thermo Fisher, Darmstadt, Germany). Gene expression levels were normalized to *Ubc*. Results were quantified using the comparative Ct method.

### Quantification and statistical analyses

All data were analyzed using GraphPad Prism version 8.0 with the exception of microbiota (16S rRNA) data. Continuous data were analyzed for normality using the D’Agostino and Pearson test and Kolmogorov–Smirnov test. When normality was confirmed, a two-way analysis of variance (ANOVA) or mixed model (when data points were missing) was used to identify statistically significant differences. When normality was not confirmed, a Kruskal–Wallis with Dunn’s correction for multiple comparisons was used. Where possible, paired or repeated measures were prioritized and indicated using line graphs. In cases where this was not possible, biospecimens collected at termination were used and indicated by grouped data (column/bar graphs). In all cases, *P* < 0.05 was considered statistically significant.

## Results

This study was performed as two distinct experiments: (1) dose finding study to identify the optimal dose of melphalan able to induce moderate, self-limiting gut toxicity, and (2) dose validation study to explore disease mechanisms. This study has been reported in accordance with the ARRIVE guidelines for transparent preclinical reporting. Baseline characteristics of the dose validation study were compared between control and melphalan groups (Table S1). There were no statistically significant differences in baseline body weight, body temperature, plasma citrulline, food intake or water intake between vehicle control animals and melphalan-treated rats.

### Intravenous melphalan induces a dose-dependent phenotype characterized by weight loss, diarrhea and mucosal injury

**Fig. 1:**
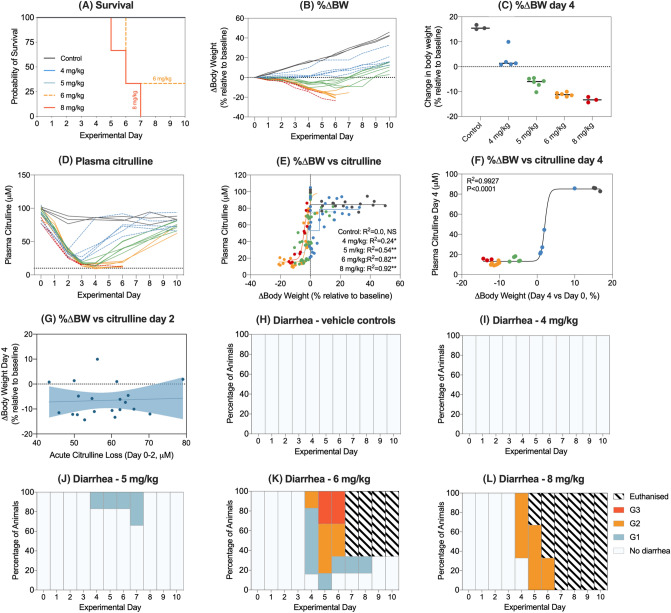
Dose finding study. To determine the optimal dose of melphalan, rats were treated with 4, 5, 6, and 8 mg/kg intravenous melphalan and clinical measures of gastrointestinal mucositis assessed for 10 days. Doses of 6 and 8 mg/kg caused unaccepted morbidity and mortality (**a**/**b**). 5 mg/kg melphalan-induced moderate, self-limiting disease. Weight loss at day 4 was dose-dependent (**c**) and correlated with plasma citrulline (**d**–**f**). Hypocitrullinemia in first 2 days after melphalan was independent of dose and did not correlate with acute weight loss (**g**). Diarrhea severity was dose-dependent (**h**–**l**). Non-linear regression analyses with Pearson’s correlation analysis was performed for all association plots

All rats received a single dose of melphalan at the designated body weight-dependent dose (4–8 mg/kg dose finding). Melphalan caused severe toxicity at doses of 6 mg/kg and 8 mg/kg with 100% mortality observed in rats treated with the highest dose (8 mg/kg; Fig. 1a). Melphalan caused dose-dependent weight loss (Fig. 1b), with maximum weight loss observed at day 4 at self-limiting doses (Fig. 1c). Melphalan caused a rapid decrease in plasma citrulline, which was lowest at day 4 (15.25 ± 1.06 μM). Citrulline was strongly correlated with weight loss at day 4 (*R*^2^ = 0.9927, *P* < 0.0001), validating its applicability as a biomarker of gut toxicity (Fig. 1d–g). Hypocitrullinemia (< 10 μM) was evident at doses of 6 mg/kg and 8 mg/kg; however, these doses were also accompanied by unacceptable diarrhea severity and mortality (100% for 8 mg/kg, Fig. 1h–l). Melphalan administered at 5 mg/kg caused self-limiting gut toxicity, with transient grade 1 diarrhea peaking at day 7 (Fig. 1j).

### Five mg/kg intravenous melphalan causes a biphasic, self-limiting clinical phenotype accompanied by fever and severe neutropenia

**Fig. 2:**
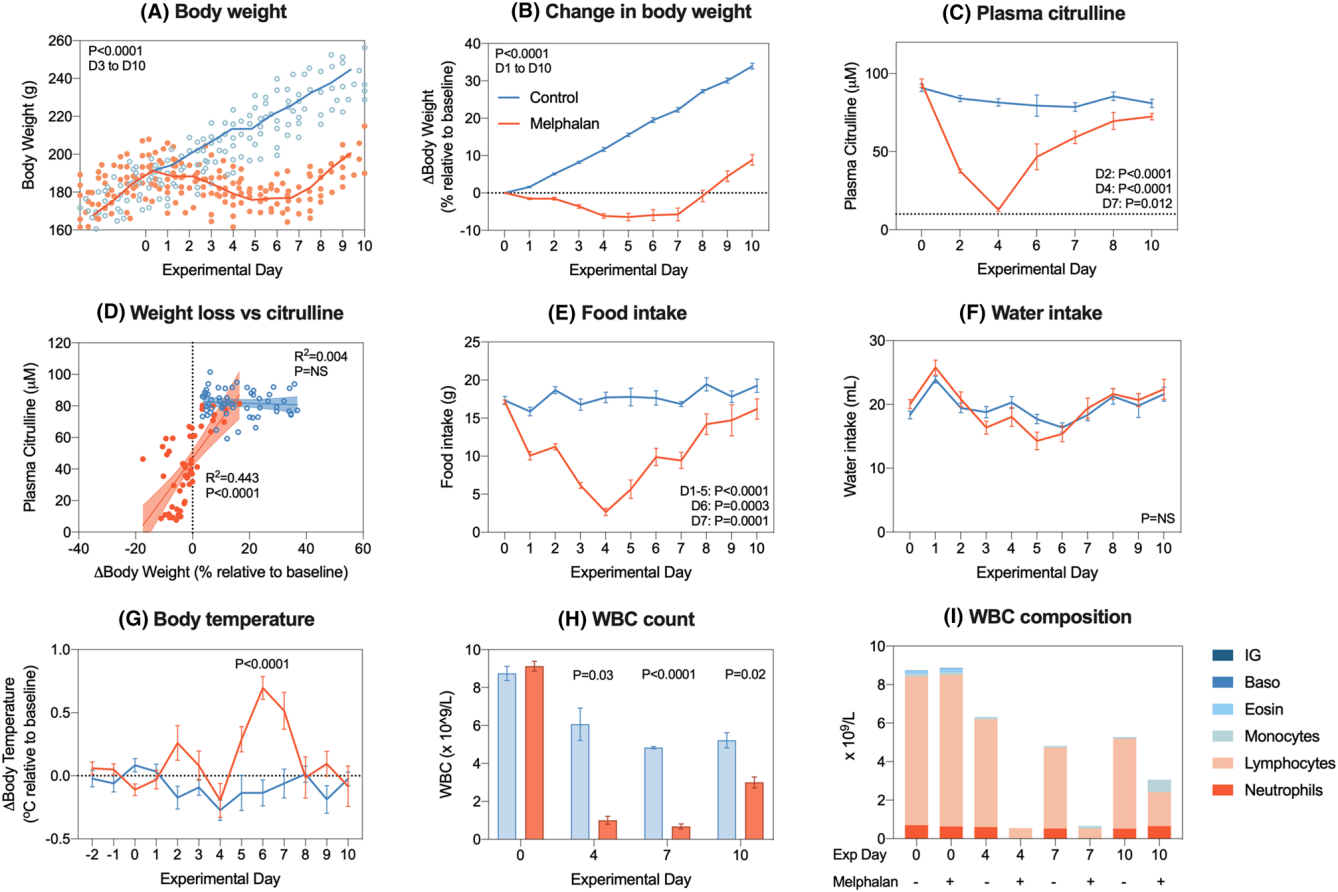
Dose validation study with 5 mg/kg intravenous melphalan. Melphalan caused transient weight loss (**a**–**b**) and hypocitrullinemia (**c**), with a strong correlation between melphalan-induced weight loss and citrulline (**d**). Melphalan caused anorexia (**e**) but did not affect water intake (**f**). Body temperature spiked 6 days post-melphalan (**g**) coinciding with white blood cell nadir (**h**–**i**). Data represented as individual values (**a**, **d**), mean ± SEM (**b**–**h**, excluding **d**) or stacked means (**i**). Simple linear regression analysis (± 95% CI) with Pearson’s correlation was performed for all association plots

Melphalan at 5 mg/kg caused significant weight loss compared to vehicle control animals (− 6.4 ± 1.01% relative to baseline, *P* < 0.0001 Fig. 2a/b). Significant decreases in citrulline were evident on day 2 (84.1 ± 1.9 μM vs 37.6 ± 1.3 μM, *P* < 0.0001), day 4 (81.5 ± 2.4 μM vs 12.8 ± 1.24 μM, *P* < 0.0001) and day 7 (78.6 ± 2.7 μM vs 59.2 ± 14.0 μM, *P* = 0.012), returning to baseline by day 10 (Fig. 2c). In melphalan-treated animals, weight loss and citrulline were moderately correlated (*R*^2^ = 0.433, *P* < 0.0001). Melphalan also caused a significant reduction in food consumption, which was significant from day 1 to 7 (Fig. 2e); however, this was not accompanied by any change in water intake (Fig. 2f).

To ensure clinical parallels, we also evaluated body temperature and white blood cell (WBC) dynamics post-melphalan treatment. Body temperature spiked at day 6 in melphalan-treated animals (− 0.14 ± 0.10 °C vs + 0.70 ± 0.09 °C, *P* < 0.0001, Fig. 2g). Melphalan also induced severe leucopenia, with a significant decrease in total WBC count at day 4 (6.1 ± 0.8 × 10^9^/L vs 1.0 ± 0.2 × 10^9^/L, *P* = 0.03, Fig. 2h), day 7 (4.8 ± 0.1 × 10^9^/L vs 0.7 ± 0.1 × 10^9^/L, *P* < 0.0001, Fig. 2h) and day 10 (5.2 ± 0.4 × 10^9^/L vs 3.0 ± 0.6 × 10^9^/L, *P* = 0.02, Fig. 2h). This was explained by significant ablation of neutrophils (*P* < 0.03), lymphocytes (*P* < 0.0001), monocytes (*P* = 0.01) and basophils (*P* = 0.03; Fig. 2i).

### Melphalan causes severe histopathological injury in the small and large intestine

**Fig. 3:**
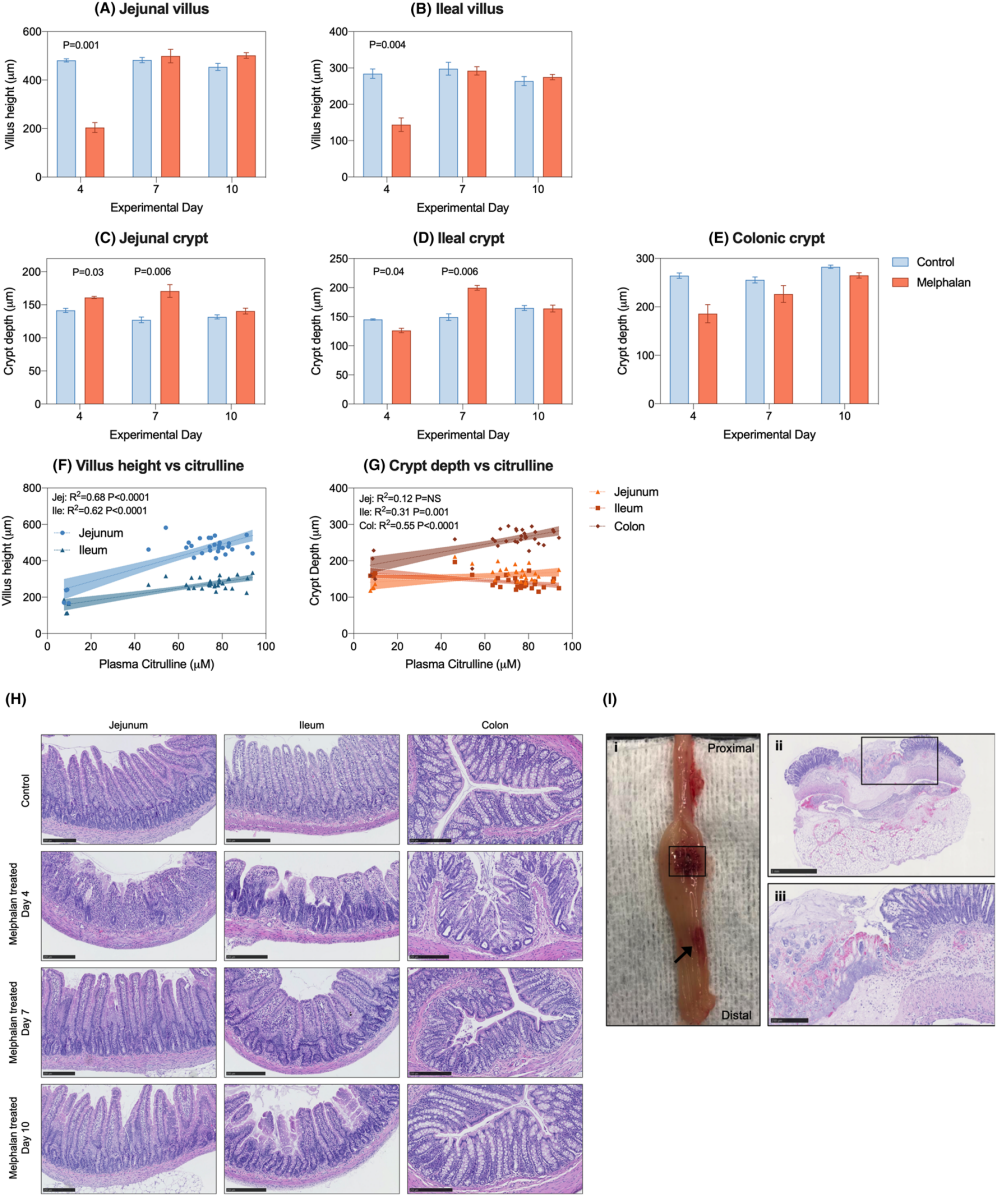
Five mg/kg intravenous melphalan causes severe anatomic derangement which correlates with plasma citrulline. Villus length (**a**, **b**) and crypt depth (**c**–**e**) were assessed at day 4, 7 and 10 and were correlated with matched plasma citrulline values (**f**–**g**). Panel **h** shows representative images of intestinal architecture. Panel **i** shows colonic perforation observed in one rat 7 days post-melphalan, with areas of frank ulceration (arrow) and total destruction of mucosa, submucosa and muscularis (**i** ii/iii). Data presented as mean ± SEM (**a**–**e**). All scale bars show 250um with the exception of panel **i** (ii) which shows 1 mm. Simple linear regression analyses (± 95% CI) with Pearson’s correlation analysis was performed for all association plots

Melphalan caused severe architectural injury in the small and large intestine, characterized by severe villus blunting/atrophy and crypt degeneration at day 4 (Fig. 3). Villus height was significantly decreased in melphalan-treated animals compared to controls in the jejunum (481.1 ± 6.5 μm vs 204.3 ± 20.3 μm, *P* = 0.001, Fig. 3a) and ileum (284.3 ± 12.7 μm vs 143.8 ± 18.6 μm, *P* = 0.004, Fig. 3b). Crypt depth was significantly increased in the jejunum at day 4 (141.6 ± 3.2 μm vs 161.0 ± 1.7 μm, *P* = 0.03, Fig. 3c) and day 7 (127.2 ± 4.3 μm vs 170.9 ± 9.7 μm, *P* = 0.006, Fig. 3c). In contrast, crypt depth in the ileum was significantly decreased at day 4 (145.2 ± 1.1 μm vs 126.3 ± 3.8 μm, *P* = 0.04, Fig. 3d) in melphalan-treated rats compared to controls, while an increase was observed at day 7 (149.4 ± 5.6 μm vs 199.7 ± 4.1 μm, *P* = 0.006, Fig. 3d). No significant changes in crypt depth were observed for the colon (Fig. 3e).

To further validate citrulline as a surrogate marker of histopathological parameters of gut toxicity (gold-standard assessment), we evaluated the association between citrulline and villus/crypt height/depth. Strong correlations were observed between villus height and citrulline for both the jejunum and ileum (*R*^2^ = 0.68, *R*^2^ = 0.62, respectively, *P* < 0.0001, Fig. 3f). Significant, but less robust correlations were observed for citrulline and crypt depth for the ileum and colon (*R*^2^ = 0.31, *P* = 0.001; *R*^2^ = 0.55, *P* < 0.0001, respectively, Fig. 3g).

**Fig. 4:**
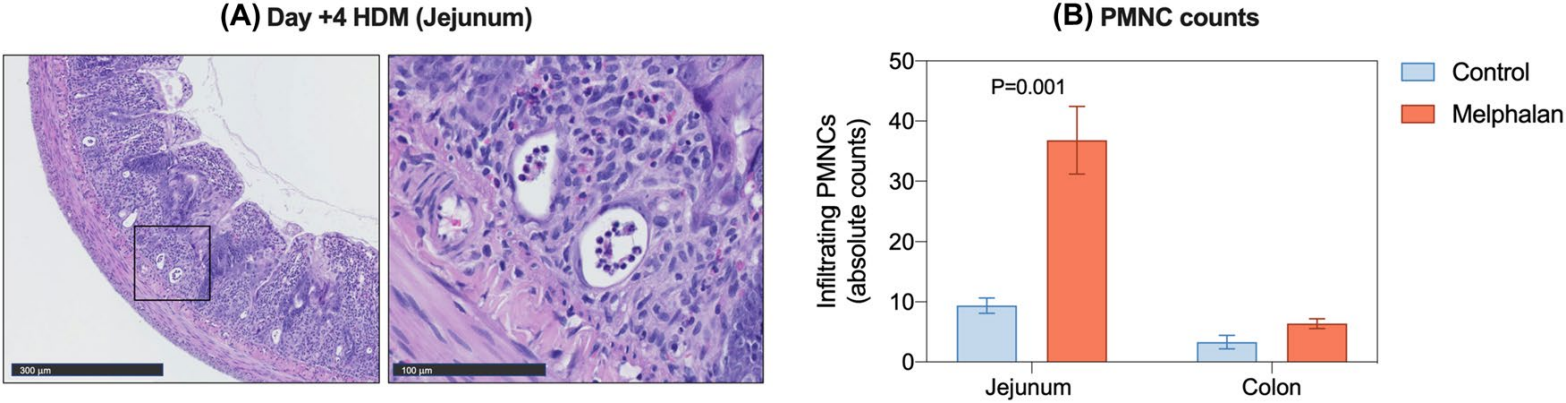
Melphalan-induced gastrointestinal injury is characterized by infiltration of polymorphic nuclear cells (PMNC) in the jejunum. (**a**) Representative images of the jejunum of melphalan-treated rats showing tissue destruction and inflammatory infiltrate. (**b**) Quantification of PMNC infiltrate in the jejunum and colon. Data presented as mean ± SEM

In addition to villus blunting and changes in crypt depth, melphalan caused severe architectural derangement in the small and large intestine, characterized by villus fusion and crypt ablation (Fig. 3h), with evidence of gross colonic pathology seen at day 7 [Fig. 3i (i)]. Histological analysis of a macroscopically evident colonic perforation showed complete destruction of the mucosa, submucosa and muscularis layer [Fig. 3i (ii)], with pseudomembrane development and visible inflammatory infiltrate [Fig. 3i (iii)]. Infiltration of polymorphic nuclear cells (PMNC) was also elevated in the jejunum of melphalan-treated rats (Fig. 4).

### Melphalan-induced microbiota disruption is characterized by pathogen expansion and SCFA deficits

**Fig. 5:**
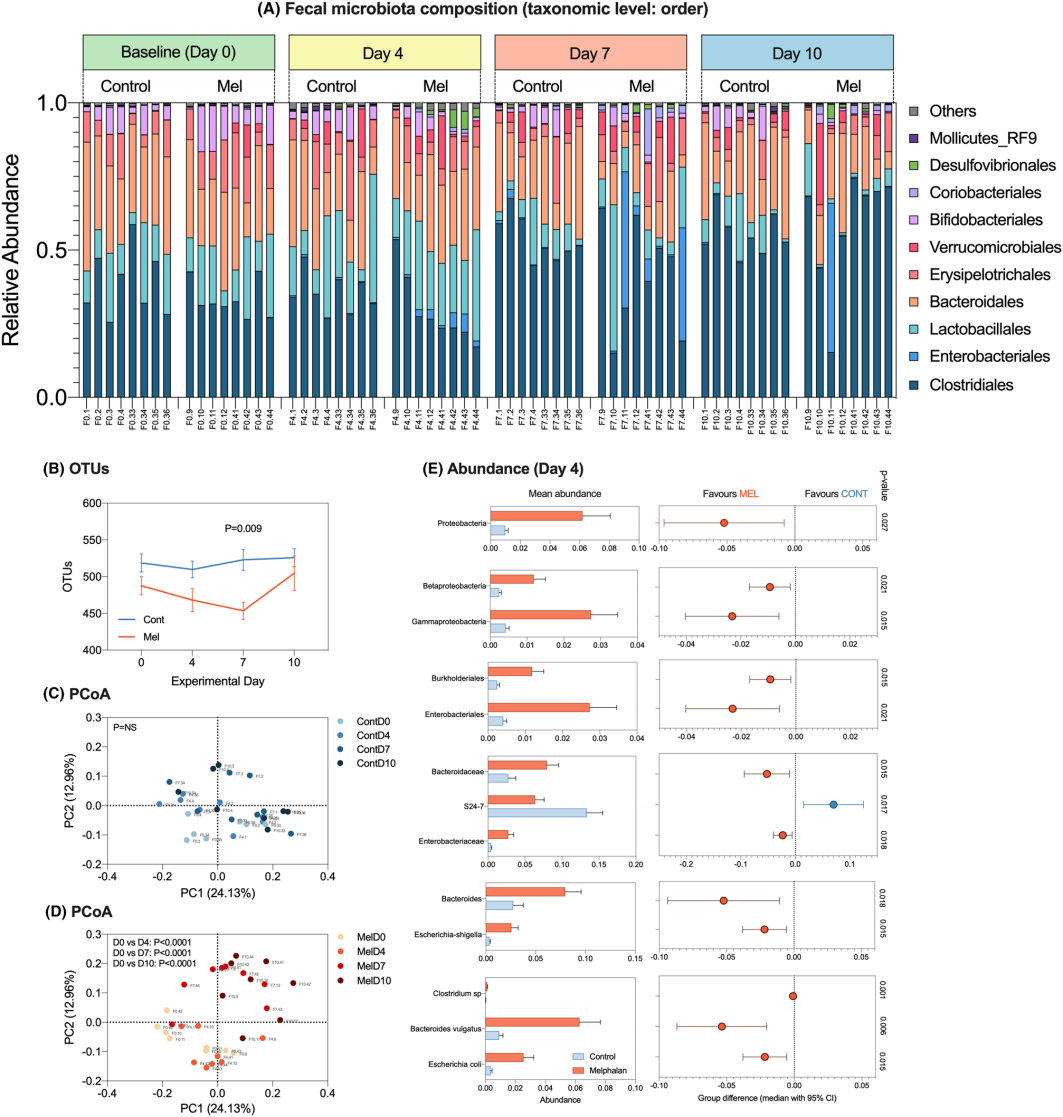
Melphalan-induced dysbiosis is characterized by expansion of enteric pathogens. Panel **a** showed relative abundance for individual animals assessed longitudinally over experimental time course. Data are shown at taxonomic level of order. OTUs were significantly decreased at day 7 post-melphalan (**b**), with principle component analyses demonstrating significant shifts in the microbiota post-melphalan that were unable to resolve (**c**–**d**). Panel **e** shows significantly altered taxa (mean ± SEM) at day 4 and median difference between groups (median ± 95% CI)

16S rRNA-gene analysis was performed in fecal samples collected longitudinally (control and melphalan, *N* = 8 per group) on day 0, 4, 7 and 10. Melphalan-induced significant disruption of the fecal microbiota that failed to recover during the 10-day experimental period (Fig. 5). Compositionally, there was a shift towards a Firmicute-dominated microbiota with expansion of pathogenic taxa largely belonging to the Proteobacteria phylum (Fig. 5a) which was not observed in control animals. This was accompanied by a decrease in the number of operational taxonomic units (OTUs), an indicator of microbial richness, which was significantly decreased 7 days post-melphalan compared to controls (*P* = 0.009, Fig. 5b). There were no significant changes in alpha diversity parameters, including Chao1, Simpson index and Shannon’s index in both control and melphalan-treated rats (data not shown). Principle component analyses showed no change in beta diversity of control animals (Fig. 5c). In contrast, significant changes were evident at all time points in melphalan-treated animals compared to baseline (*P* < 0.0001, Fig. 5d). These were also significant compared to controls at day 4 (*P* = 0.021) and day 7 (*P* = 0.015, data not shown).

While beta diversity and richness were most profoundly affected at day 7, expansion of pathogens was most significant at day 4 post-melphalan (Fig. 5e), with significant increases in the abundance of *Proteobacteria* (*P* = 0.027), *Betaproteobacteria* (*P* = 0.021), *Gammaproteobacteria* (*P* = 0.015), *Burkholderiales* (*P* = 0.015), *Enterobacteriales* (*P* = 0.021), *Bacteroidaceae* (*P* = 0.015), *Enterobacteriaceae* (*P* = 0.018), *Bacteroides* (*P* = 0.018), *Escherichia-shigella* (*P* = 0.015), *Clostridium sp*. (*P* = 0.001), *B. vulgatus* (*P* = 0.006) and *E. coli* (*P* = 0.015). This was accompanied by a detectable decrease in *Muribaculum (S24-7)* in melphalan-treated rats compared to controls (*P* = 0.017).

**Fig. 6:**
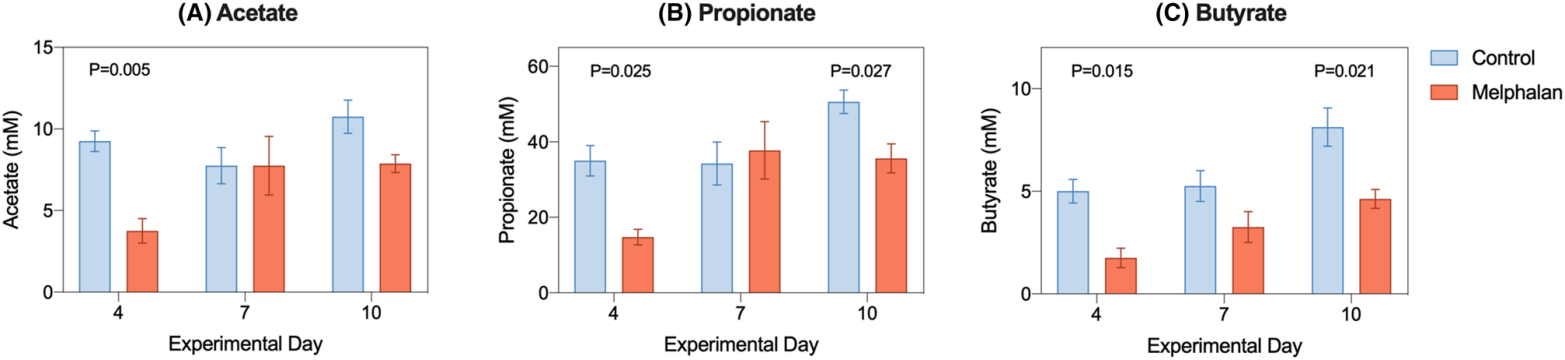
Cecal short chain fatty acid (SCFA) profiles change following intravenous melphalan. Acetate (**a**), propionate (**b**) and butyrate (**c**) were all decreased at day 4 following melphalan. Data represented as mean ± SEM

To determine the functional impact of microbial changes, short chain fatty acids (SCFAs) were quantified in cecal samples collected at termination at all time points. Significant decreases in acetate (9.25 ± 0.63 mM vs 3.75 ± 0.75 mM, *P* = 0.005, Fig. 6a), propionate (35.12 ± 4.02 mM vs 14.75 ± 2.05 mM, *P* = 0.025, Fig. 6b) and butyrate (5.00 ± 0.58 mM vs 1.75 ± 0.47 mM, *P* = 0.015, Fig. 6c) were observed at day 4. Significant deficits in propionate and butyrate were also observed at day 10 (*P* = 0.027, *P* = 0.021, respectively).

Orally administered FITC-dextran was used to assess epithelial barrier permeability at all terminal time points. While no significant changes were observed, the highest concentration of plasma FITC-dextran was observed on day 7 post-melphalan treatment (Figure S2). At this time point, we also identified one case of positive blood culture (*E. coli*). All other blood cultures were negative.

### Melphalan-induced ileal injury results in bile acid malabsorption and decreased plasma primary to secondary bile acid ratios

**Fig. 7:**
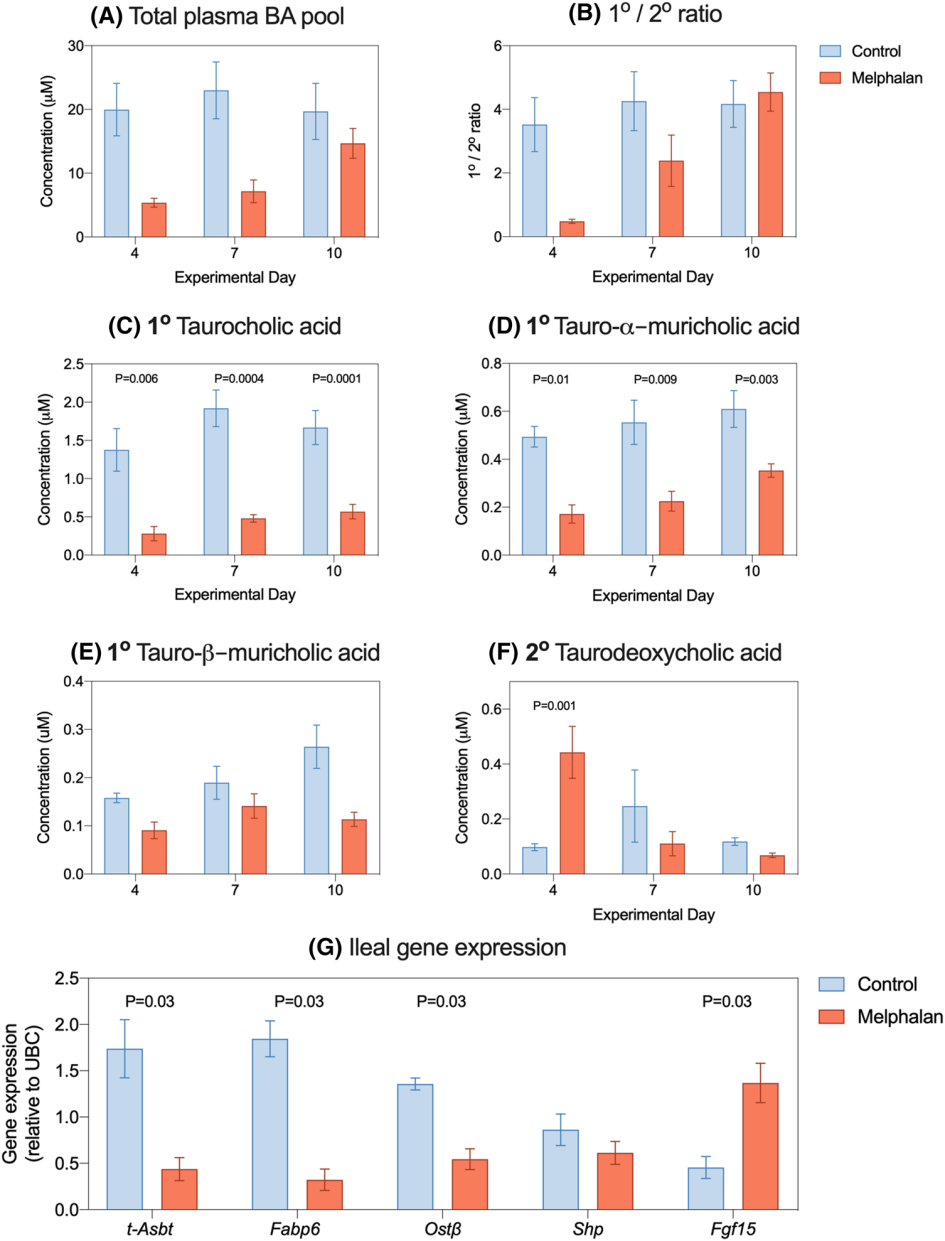
Bile acid malabsorption is seen in melphalan-treated rats. Bile acids were assessed in plasma isolated from whole blood collected at termination. No significant decreases were seen in total bile acid pool (**a**) or the ratio of primary to secondary bile acids (**b**). Significant decreases were identified for taurocholic acid (**c**) and tauro-alpha-muricholic acid (**d**) but not for tauro-beta-muricholic acid (**e**). Taurodeoxycholic acid was increased in plasma on day 4 (**f**). Ileal gene expression was performed for downstream bile acid targets at day 4 (**g**). Data shown as mean ± SEM

Plasma bile acids profiles were analyzed at termination on day 4, 7 and 10. Total plasma bile acid concentrations and primary/secondary ratios were decreased in melphalan-treated animals on day 4 and 7 but this did not reach statistical significance (Fig. 7a, b). Upon analyzing individual bile acid species, the primary conjugated bile acid, taurocholic acid (TCA), was decreased at all evaluated time points post-melphalan treatment (Fig. 7c, *P* = 0.006, *P* = 0.0004, *P* = 0.0001, respectively). The rodent-specific primary bile acid tauro-alpha-muricholic acid (T-α-MCA) was decreased at all time points (Fig. 7d, *P* = 0.01, *P* = 0.003, *P* = 0.009, respectively), while beta-muricholic acid was unchanged (Fig. 7e). The secondary bile acid, deoxycholic acid (DCA), was unchanged by melphalan treatment in its unconjugated form (supplementary data) but higher as taurine conjugate at day 4 (*P* = 0.001, Fig. 7f). All other bile acid concentrations are listed in Table S2.

Ileal gene expression analysis was performed at day 4 for target genes involved in bile acid absorption and transport (Fig. 7g). Ileal expression of *t-Asbt, Fabp6 and Ostβ* was significantly lower in melphalan treatment compared to controls, suggesting lower ileal absorption of bile acids (*P* = 0.03). In contrast, farnesoid X receptor (FXR) target genes Shp and Fgf15 were unchanged and increased (*P* = 0.03), respectively.

## Discussion

The toxic properties of melphalan, mainly gut toxicity, fever and infections, currently prohibit expansion of its indication to older and less fit patients. Moreover, extensive antimicrobial drug use contributes to the growing global threat of antimicrobial resistance, disruption of gut microbiota, and increased costs.

Here, we highlight a critical advance in not only modeling HDM-induced gut toxicity, but also the mechanisms that drive acute epithelial injury and secondary infectious complications. To our knowledge, this is the only preclinical model that recapitulates the clinically relevant interplay of events including mucosal injury, neutropenia, microbial disruption and fever caused by melphalan, and thus represents a translationally robust approach for the study of HSCT-associated toxicities and evaluation of new interventions.

Intravenous melphalan (5 mg/kg) caused moderate, self-limiting gut toxicity characterized by a clinically relevant phenotype. Critically, liquid biomarkers (citrulline), microbial dynamics and toxic events mirror previously published clinical human datasets [5] and correlate with gold-standard histopathological criteria used in existing models of gut toxicity [29, 34, 35]. This reinforces the use of plasma citrulline as a minimally invasive liquid biomarker of gut toxicity, permitting repeated measures and reducing animal numbers. This highlights the applicability of this model for rapid interventional pre-screening with a greater likelihood of clinical success.

While many models have been successfully developed to understand chemotherapy-induced mucosal injury, many of these translate poorly to hematological malignancies in which mucositis is accompanied by severe neutropenia. While neutropenia has long been considered critical in driving infectious complications of HSCT, an increasing body of evidence suggests that neutropenia is not solely responsible for infectious events often seen in HSCT recipients [7, 36, 37]. Importantly, antimicrobial agents and hematopoietic growth factors have both failed to significantly reduce the incidence of infection/fever, with disruption of the host microbiota and breakdown of the mucosal barrier now considered critical factors in the initiation of blood stream infection. While no profound changes were identified in diversity and richness indices, melphalan treatment resulted in functional deficits in the microbial metabolome, indicated by short chain fatty acid (SCFA) loss, resulting in a pathogen-dominated microbiota. SCFAs serve to not only support mucosal homeostasis, but are critical in the acidification of the luminal environment; an important defense against the expansion of pathogens, particularly *Enterobacteriaceae* [38, 39]. As such, it is likely that loss of luminal SCFAs promotes self-perpetuating mucosal injury and microbial disruption that contribute to acute gut toxicity and infection risk (Fig S3).

Our model is the first to demonstrate significant expansion of pathogenic microbes following melphalan treatment, yet in the absence of antibiotics, coinciding with peak mucosal injury and inflammation and preceding fever and an isolated case of blood stream infection. Importantly, we showed significant fecal expansion of *E. coli*, which was identified by MALDI-TOF analysis of a positive blood culture. These results are consistent with reports from 2003 in which mucosal inflammation preceded bacteremia and fever [40] and align with findings demonstrating enteric domination by *Enterobacteriaceae* drastically increases the risk of bacteremia in allogeneic HSCT recipients [41, 42]. These data suggest translocation of endogenous microbes across a damaged mucosal barrier is a key driver of blood stream infection. Efforts to maintain the mucosal barrier and host microbiota are therefore warranted and may aid in reducing the over-reliance on antimicrobials. This is particularly relevant in the broader setting of HSCT, in which antibiotic-induced disruption of the microbiota has been shown to exacerbate graft-versus-host disease (GvHD) and increase mortality [12, 43].

The impact of antibiotics on the toxicity outcomes of HSCT has been the subject of intense investigation. In fact, the large majority of studies attribute microbial changes observed in HSCT to prophylactic and empirical antibiotic use (key studies summarized by Zama 2020 [44]). Here, we demonstrate that changes in the microbiota occur in the *absence* of antibiotics supporting either direct bactericidal properties of melphalan or mucosal-dependent mechanisms of dysbiosis. However, we were surprised that alpha diversity indices did not capture the changes in microbial composition, with no changes in alpha diversity seen in our model. This is in contrast to clinical observations [45] where profound diversity changes were observed. The lack of change we detected in alpha diversity may simply reflect the underpowered nature of alpha diversity indices which may have underestimated the magnitude of effect. This could be explored by applying error models adjusted for uncertainty; however, this is beyond the scope of the current paper. Alternatively, our findings may simply suggest that antibiotics are required for a detectable change in alpha diversity, with HDM inducing compositional changes only. Nonetheless, we were encouraged to see microbial changes that were consistent with findings in patients undergoing HDM which were associated with diarrhea and culture-negative fever, reiterating the use of our model to further explore the relationship between the microbiome and treatment outcomes in HSCT recipients [45].

Consistent with the findings of Tsuji et al. (2003) and Taur et al. (2012), we observed a biphasic phenotype, with peak morbidity observed at day 4 and a secondary episode at day 6–7. Acute morbidity reflects direct cytotoxic injury to the intestinal mucosa, consistent with the findings reported in other longstanding models of gut toxicity [35]. However, histopathological analysis and plasma citrulline data indicate that the mucosa of the small intestine is recovering by day 6–7. In contrast, colonic histopathological analysis identified residual injury at day 6–7, coinciding with peak intestinal permeability (FITC-dextran) suggesting a recovering, yet immature and functionally deficient mucosa that does not exert the same degree of pre-melphalan barrier control [46]. As such, following the expansion of enteric pathogens, a leaky colonic barrier then permits translocation and prompts subsequent fever. This proposed interaction aligns with higher microbial load in the distal gastrointestinal tract, and higher abundance of innate immune receptors (such as Toll-like receptor 4) which are critical in the regulation of the intestinal barrier after chemotherapy [29, 47].

In addition to the fundamental insight we provide, our data also identify new therapeutic strategies that will mitigate acute gut toxicity with the goal of preventing infection and prompting appropriate antibiotic stewardship. Of particular interest were changes in bile acid metabolism, with our findings supporting use of bile acid sequestrants consistent with their use in other gastrointestinal diseases [48].

Bile acid malabsorption (BAM) has been widely implicated in the development of diarrhea, including diarrhea caused by the tyrosine kinase inhibitor neratinib [49] and in patients with GI-GvHD [50], increasing colonic secretion of water and electrolytes and the induction of propagated contractions [51]. We identified changes in plasma bile acids and ileal gene expression following melphalan, indicative of BAM. BAM increases the pool of luminal bile acids, with our data supporting microbiota-dependent production of secondary bile acids [52]. Importantly, we observed a relative increase of fecal bacteria from the Firmicutes phylum upon melphalan treatment. It is known that Firmicutes, especially certain *Clostridia* species, are able to 7-alpha-hydroxylate bile acids thus leading to an increased secondary pool as observed in our study [53, 54]. Secondary bile acids are mucotoxic and pro-inflammatory [55], and as such, their production may serve to amplify mucosal injury. The clinical relevance of this finding is particularly compelling with BAM reported in severe cases of GvHD-related diarrhea and even bowel perforation [50, 56]. Bile acid sequestrants (colesevelam) may offer benefits in both the prevention of acute diarrhea (caused by motility/secretory mechanisms) as well as the prevention of colonic permeability responsible for bacterial translocation. Coleveselam has already been deemed safe in oncology cohorts and has demonstrated clinical efficacy in treatment neratinib-induced diarrhea [49, 57].

In summary, we have developed the first translational model of HDM-induced gut toxicity that reflects the clinical phenotype of HSCT-associated toxicities. Our results reinforce the multi-factorial nature of gut toxicity and its symptomology, with direct cytotoxic injury an important initiating factor in establishing various cascades of injury. This involves neutrophil recruitment, inflammation, microbial changes and bile acid disruption, each of which are likely to contribute to bacterial translocation and subsequent blood stream infection. Critically, we suggest that this sequence of events only stops when stem cells repopulate the intestinal lining, renewing niches to support the host’s commensal microbiota. Replenished SCFA production and normalized enterohepatic circulation then aid in re-establishing a functional barrier. These mechanistic insights highlight that systemic reactions such as fever are a form of collateral damage resulting from breakdown of the intestinal mucosal/luminal environments. Efforts to intervene early in these sequelae are therefore of great clinical significance.

## Supplementary Information

Below is the link to the electronic supplementary material.

Supplementary file1 (DOCX 18 KB)

Supplementary file2 (DOCX 22 KB)

Supplementary file3 (PDF 24 KB)

Supplementary file4 (TIFF 119 KB)

Supplementary file5 (PNG 167 KB)

## Data Availability

All data will be made freely available upon request. Requests must be made to corresponding author Dr Hannah Wardill (hannah.wardill@adelaide.edu.au).
