# Supplementary material for: Translational model of melphalan-induced gut toxicity reveals drug-host-microbe interactions that drive tissue injury and fever
Source: Cancer Chemother Pharmacol. 2021 Apr 20;88(2):173–88. doi: 10.1007/s00280-021-04273-7 (PMC8236460; doi:10.1007/s00280-021-04273-7)
Supplement: Supplementary file 2 — Supplementary file2 (DOCX 22 KB) [file 280_2021_4273_MOESM2_ESM.docx]

Supplementary Information: Tables

| **Table S1: Baseline characteristics (mean ± SEM)** | | | |
| --- | --- | --- | --- |
|  | Vehicle control | 5 mg/kg melphalan | P value |
| Body weight (g) | 177.1 ± 4.4 | 182.4 ± 4.2 | 0.42 |
| Body temperature (^o^C) | 37.24 ± 0.05 | 37.05 ± 0.04 | 0.06 |
| Daily food intake (g) | 17.32 ± 0.53 | 17.14 ± 0.25 | 0.76 |
| Daily water intake (g) | 18.36 ± 9.62 | 20.07 ± 0.70 | 0.08 |
| Plasma citrulline (g) | 90.89 ± 2.37 | 93.78 ± 2.82 | 0.44 |

| **Table S2: Plasma bile acid concentrations** | | | | | | | |
| --- | --- | --- | --- | --- | --- | --- | --- |
|  |  |  | **Control** | | **Melphalan** | |  |
|  |  | **Day** | **Mean** | **SEM** | **Mean** | **SEM** | **Sig** |
| Total BA pool | | 4 | 19.97 | 4.12 | 5.36 | 0.69 |  |
|  |  | 7 | 22.99 | 4.45 | 7.16 | 1.78 |  |
|  |  | 10 | 19.69 | 4.40 | 14.68 | 2.34 |  |
| Primary BA | CA | 4 | 10.02 | 1.69 | 0.30 | 0.12 | * |
|  |  | 7 | 10.62 | 1.40 | 2.68 | 1.27 | * |
|  |  | 10 | 9.82 | 2.56 | 7.44 | 1.39 |  |
|  | GCA | 4 | 0.12 | 0.04 | 0.07 | 0.03 |  |
|  |  | 7 | 0.24 | 0.09 | 0.13 | 0.03 |  |
|  |  | 10 | 0.20 | 0.05 | 0.12 | 0.02 |  |
|  | TCA | 4 | 1.38 | 0.28 | 0.28 | 0.09 | ** |
|  |  | 7 | 1.92 | 0.24 | 0.48 | 0.05 | *** |
|  |  | 10 | 1.67 | 0.22 | 0.57 | 0.09 | *** |
|  | CDCA | 4 | 1.72 | 0.72 | 4.00 | 0.24 |  |
|  |  | 7 | 1.59 | 0.28 | 4.00 | 0.28 |  |
|  |  | 10 | 1.71 | 0.47 | 7.00 | 1.31 |  |
|  | GCDCA | 4 | 0.03 | 0.00 | 0.01 | 0.01 |  |
|  |  | 7 | 0.03 | 0.01 | 0.01 | 0.01 |  |
|  |  | 10 | 0.03 | 0.01 | 0.02 | 0.00 |  |
|  | TCDCA | 4 | 0.17 | 0.01 | 0.19 | 0.01 |  |
|  |  | 7 | 0.22 | 0.05 | 0.08 | 0.02 | ** |
|  |  | 10 | 0.24 | 0.04 | 0.13 | 0.01 | ** |
|  | α-MCA | 4 | 1.29 | 0.46 | 0.35 | 0.15 |  |
|  |  | 7 | 1.28 | 0.12 | 0.37 | 0.16 |  |
|  |  | 10 | 1.39 | 0.37 | 1.34 | 0.21 |  |
|  | T-α-MCA | 4 | 0.49 | 0.04 | 0.17 | 0.04 | ** |
|  |  | 7 | 0.55 | 0.09 | 0.23 | 0.04 | ** |
|  |  | 10 | 0.61 | 0.08 | 0.35 | 0.03 | ** |
|  | β-MCA | 4 | 0.71 | 0.26 | 0.93 | 0.51 |  |
|  |  | 7 | 0.83 | 0.16 | 0.68 | 0.27 |  |
|  |  | 10 | 0.92 | 0.28 | 0.97 | 0.17 |  |
|  | T-β  -MCA | 4 | 0.16 | 0.01 | 0.09 | 0.02 |  |
|  |  | 7 | 0.19 | 0.03 | 0.14 | 0.03 |  |
|  |  | 10 | 0.26 | 0.05 | 0.11 | 0.01 | * |
| Secondary BA | DCA | 4 | 0.47 | 0.26 | 1.13 | 0.57 |  |
|  |  | 7 | 0.34 | 0.08 | 0.16 | 0.03 |  |
|  |  | 10 | 0.43 | 0.10 | 0.28 | 0.06 |  |
|  | GDCA | 4 | 0.01 | 0.01 | 0.06 | 0.01 | * |
|  |  | 7 | 0.02 | 0.01 | 0.01 | 0.01 |  |
|  |  | 10 | 0.03 | 0.01 | 0.02 | 0.01 |  |
|  | TDCA | 4 | 0.10 | 0.01 | 0.44 | 0.09 | ** |
|  |  | 7 | 0.25 | 0.13 | 0.11 | 0.04 |  |
|  |  | 10 | 0.12 | 0.01 | 0.07 | 0.01 |  |
|  | LCA | 4 | 0.03 | 0.01 | 0.06 | 0.02 |  |
|  |  | 7 | 0.02 | 0.01 | 0.00 | 0.00 |  |
|  |  | 10 | 0.02 | 0.01 | 0.01 | 0.00 |  |
|  | ω-MCA | 4 | 1.22 | 0.51 | 2.82 | 0.72 |  |
|  |  | 7 | 1.49 | 0.38 | 1.62 | 0.46 |  |
|  |  | 10 | 1.81 | 0.41 | 1.50 | 0.19 |  |
|  | HDCA | 4 | 0.08 | 0.04 | 0.05 | 0.02 |  |
|  |  | 7 | 0.11 | 0.02 | 0.05 | 0.02 |  |
|  |  | 10 | 0.14 | 0.04 | 0.18 | 0.04 |  |
