# Supplementary figures and images for: Translational model of melphalan-induced gut toxicity reveals drug-host-microbe interactions that drive tissue injury and fever
Source: Cancer Chemother Pharmacol. 2021 Apr 20;88(2):173–88. doi: 10.1007/s00280-021-04273-7 (PMC8236460; doi:10.1007/s00280-021-04273-7)

# Dose-finding (N=24)

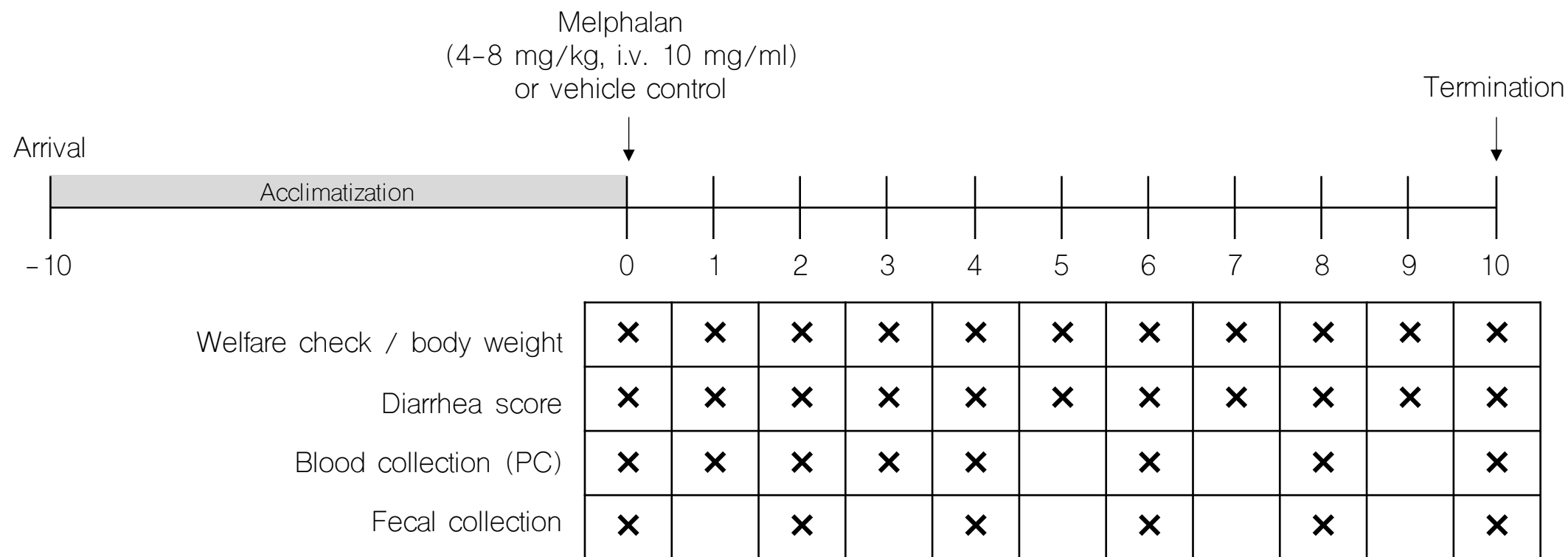

# Dose validation (N=48)

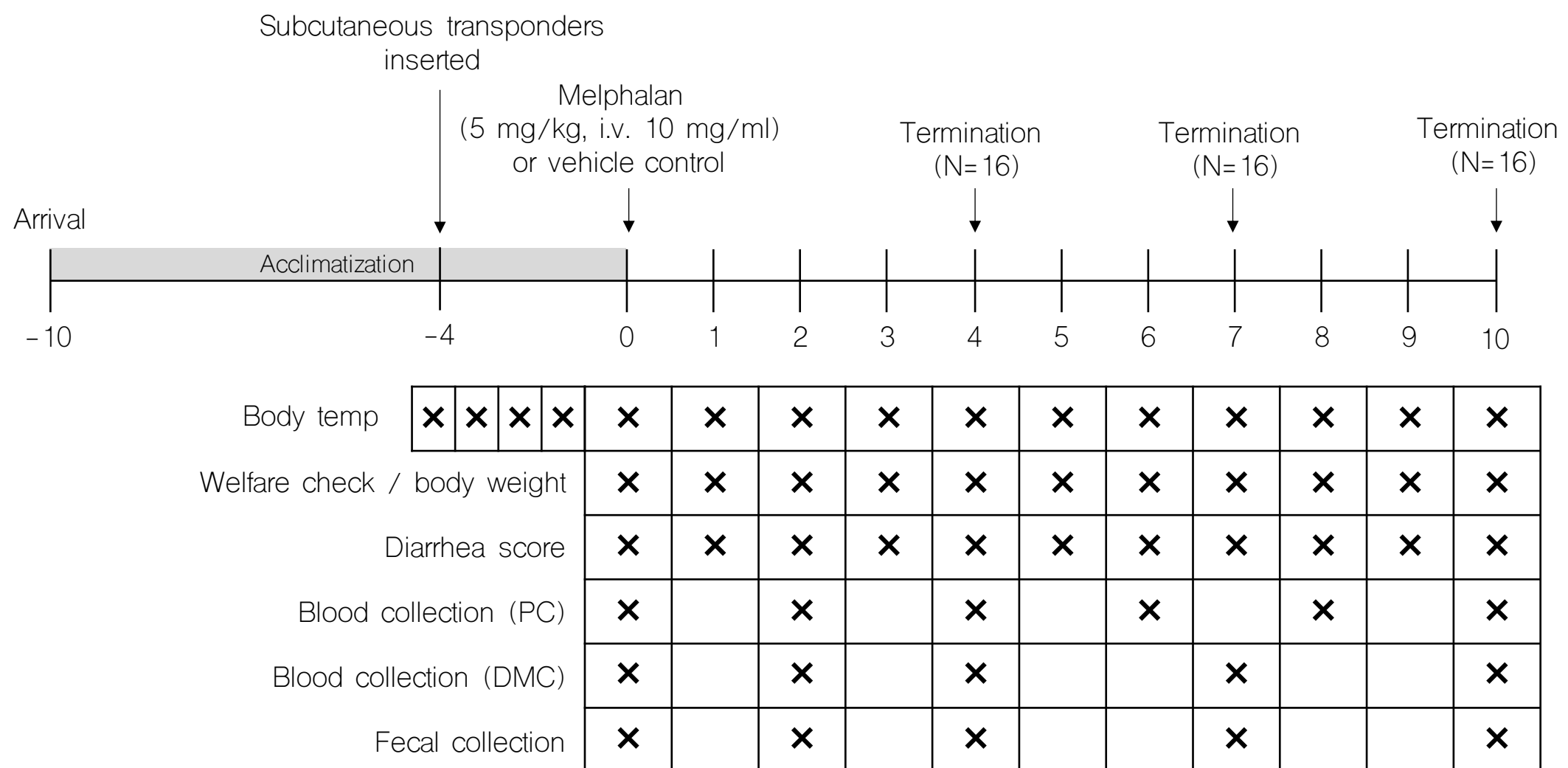

Supplement: Supplementary file 3 — Supplementary file3 (PDF 24 KB) [file 280_2021_4273_MOESM3_ESM.pdf]

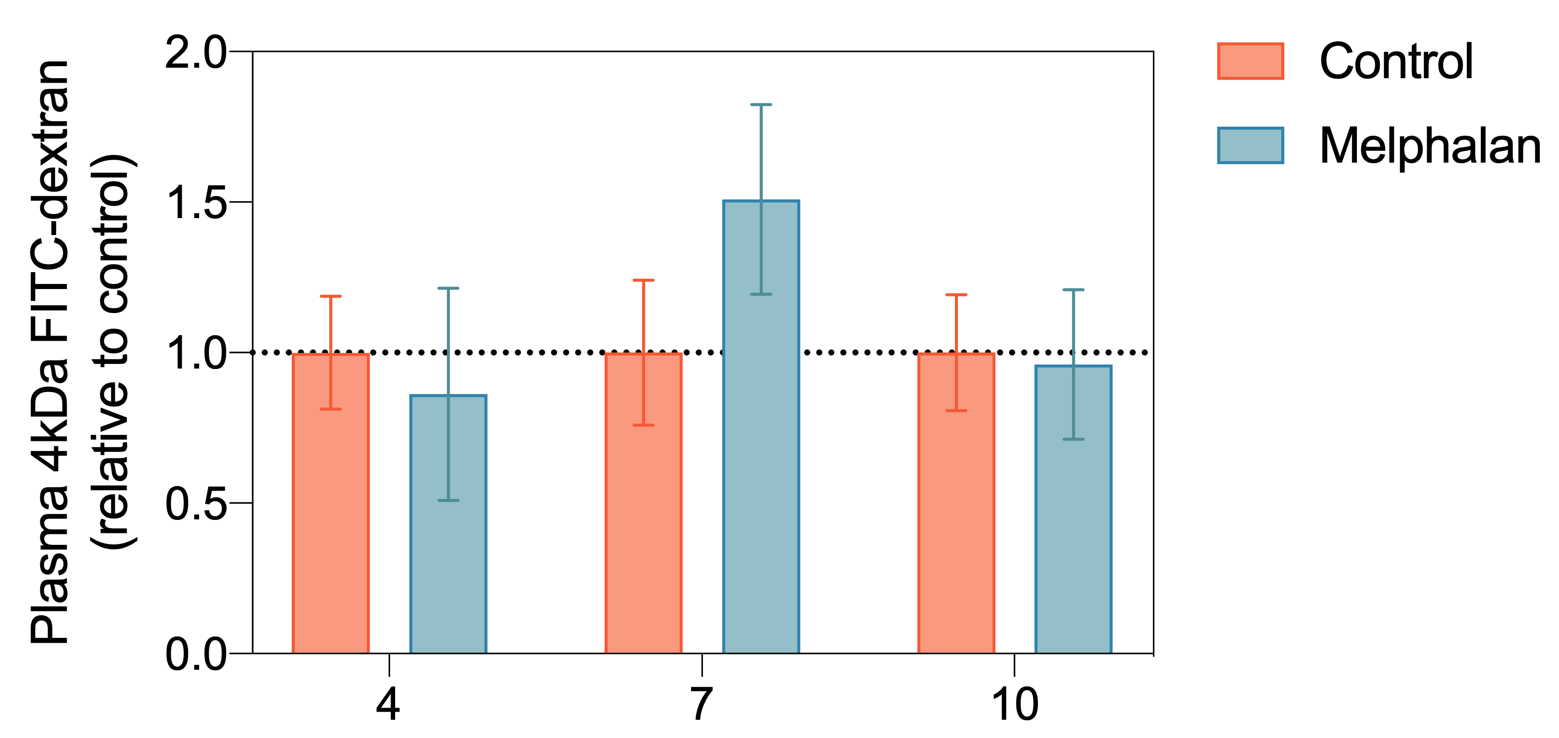

Supplement: Supplementary file 4 — Supplementary file4 (TIFF 119 KB) [file 280_2021_4273_MOESM4_ESM.tiff]

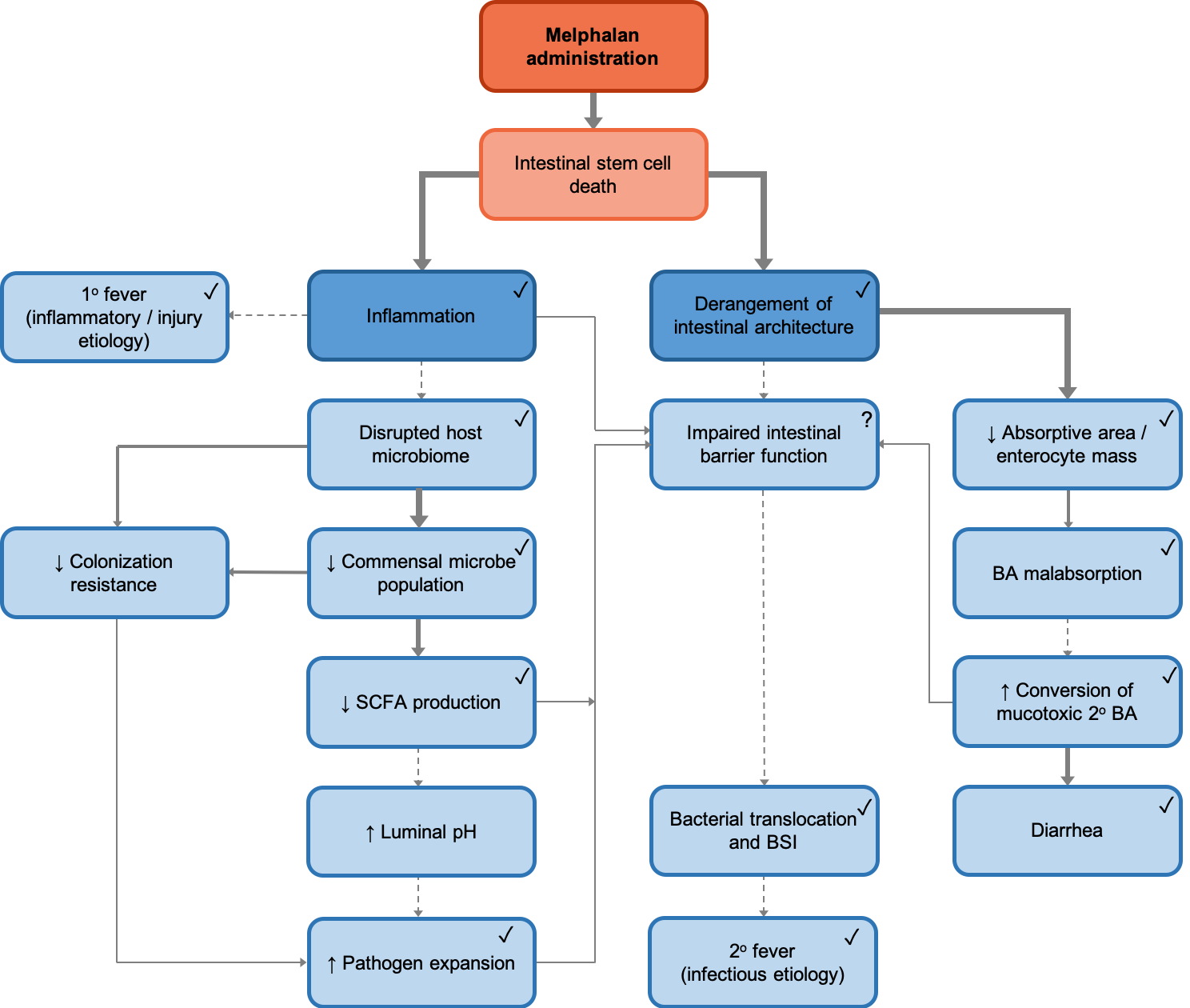

Supplement: Supplementary file 5 — Supplementary file5 (PNG 167 KB) [file 280_2021_4273_MOESM5_ESM.png]
